# Preparation, acid modification and catalytic activity of kaolinite nanotubes in α-pinene oxide isomerization

**DOI:** 10.1039/d4ra03777d

**Published:** 2024-08-16

**Authors:** Alexander Yu. Sidorenko, Tatiana V. Khalimonyuk, Behzodjon D. Mamatkodirov, Yoldosh Yu. Yakubov, Atte Aho, Tatiana V. Sviridova, Tatiana F. Kouznetsova, Bobirjon Z. Adizov, Aziz B. Ibragimov, Dmitry Yu. Murzin, Yanlong Gu, Vladimir E. Agabekov

**Affiliations:** a Institute of Chemistry of New Materials of National Academy of Sciences of Belarus 220084, Skaryna str, 36 220141 Minsk Belarus Sidorenko@ichnm.by +375 17 379 63 08; b Institute of General and Inorganic Chemistry of the Academy of Sciences of the Republic of Uzbekistan 100170, Mirzo-Ulugbek str., 77-a Tashkent Uzbekistan; c Åbo Akademi University Henriksgatan 2 20500 Turku/Åbo Finland dmurzin@abo.fi +358 2 215 4985; d Faculty of Chemistry, Belarusian State University 220050 Leningradskaya str., 14 Minsk Belarus; e Institute of General and Inorganic Chemistry of NAS of Belarus 220072, Surganov str, 9/1 Minsk Belarus; f Huazhong University of Science and Technology 103 7 Luoyu Road, Hongshan District Wuhan 430074 China

## Abstract

In this work kaolinite nanotubes (KNT) were obtained from commercial kaolin AKF-78 (Uzbekistan) by starting material sequential intercalation by DMSO and methanol, followed by treatment with a cetyltrimethylammonium chloride solution. Acid functionalization of KNT for catalytic applications was successfully performed for the first time using a two-step treatment with piranha solution (H_2_SO_4_–H_2_O_2_), which resulted in the removal of organic impurities as synthetic artifacts and an increase in specific surface area by 3.9 times (up to 159 m^2^ g^−1^), pore volume by 1.5 times (0.23 cm^3^ g^−1^) and acidity by 4.1 times (49 μmol g^−1^). The values of the porous structure parameters and concentration of acid sites in processed kaolinite nanotubes practically corresponded to those for natural halloysite nanotubes (HNT) modified in the same way. Both types of materials demonstrated catalytic activity in the model reaction of α-pinene oxide isomerization in various solvents, including green ones, with selectivity to *trans*-carveol up to 55–57% and campholenic aldehyde of 50–51%, depending on the medium used. A satisfactory correlation between solvent polarity and selectivity was also observed. To the best of our knowledge, this is the first example of using modified kaolinite nanotubes *per se* as a catalyst. Overall, treatment of KNT with piranha solution provides not only catalytic activity but also the opportunity for further functionalization and application of these nanomaterials.

## Abstract 2

Commercial kaolin after sequential intercalation by DMSO and methanol was treated with CTACl solution giving kaolinite nanotubes. After acid functionalization with the piranha solution the catalyst was applied in α-pinene oxide isomerization.

## Graphical abstract

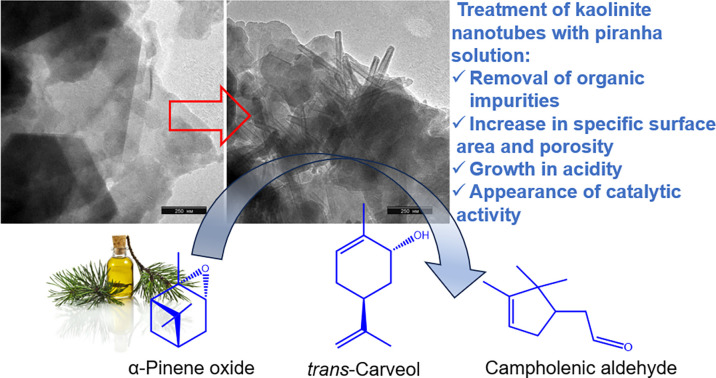

## 1. Introduction

A representative of natural materials with a nanotubular structure is halloysite, a mineral of the kaolinite group, the layer of which consists of tetrahedral Si–O and octahedral Al–O networks.^1^ Halloysite nanotubes (HNT) can be used as basis carriers for biologically active compounds, catalysts and adsorbents.^1–3^ For example, acid-modified halloysite is an effective catalyst for the Prins reaction of unsaturated compounds with aldehydes for the synthesis of products with benzopyran,^4^ tetrahydropyran,^5^ isobenzofuran^6^ and other types of structures.^7^ There are examples of the use of functionalized HNT in the Biginelli reaction for synthesis of 3,4-dihydropyrimidinones,^8^ preparation of 5-ethoxymethylfurfural as a biofuel component,^9^ and in a number of other transformations.^2,3^

**Fig. 1:**
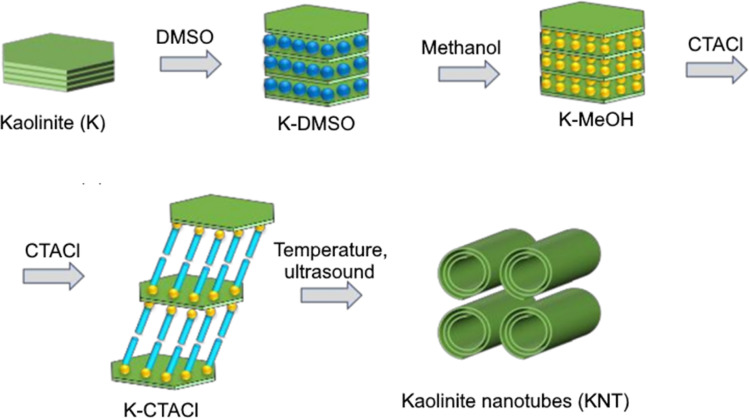
Schematic illustration for preparation of KNT from kaolin (modified from ref. 13).

In recent years, as an alternative to natural halloysite, synthetic nanotubes have been studied, which can be obtained from kaolinite by sequential introduction (intercalation) of reagents into its interlayer space with subsequent directed deformation of the layers by chemical and ultrasonic treatment,^10–15^ which can be illustrated in Fig. 1.

Thus, the intercalation of dimethyl sulfoxide (DMSO), methanol (MeOH) and alkyltrimethylammonium salts into the kaolinite structure leads to an increase in the distance between the layers and weakening of hydrogen bonds between them.^10–12^ Further heating or exposure to ultrasound of the resulting material leads to deformation (scrolling) of the aluminosilicate layers until a stable tubular shape is achieved.^10–12^

Intercalation of Chinese kaolinite with DMSO, MeOH followed by exposure to a methanol solution of cetyltrimethylammonium chloride (CTACl) for 24 h at temperatures from 30 to 100 °C led to formation of kaolinite nanotubes (KNT).^10^ KNT was prepared by stirring kaolin clay intercalated with methanol in a CTACl solution at room temperature (72 h) followed by ultrasound treatment.^11^

Treatment with methanol is one of the problematic stages in the synthesis of nanotubes, since the duration of the treatment is 7–10 days^10,11^ with the amount of reagent reaching up to 200 mL g^−1^ due to need to replace it ten times with the fresh solvent. Reducing the time of such intercalations can be achieved by using a Soxhlet extractor avoiding the need for periodic replacement of methanol, thereby reducing the solvent consumption to 20 mL g^−1^.^14^

Recently, the authors developed a method for producing nanotubes from natural kaolin (Dedovka mine, Belarus) with a length of 800–1100 nm and a diameter of 50–60 nm.^15^ The synthesis was carried out under relatively mild conditions (60–66 °C, atmospheric pressure) and low (7.0 mL g^−1^) amounts of MeOH required for their formation.

Acid functionalization of natural halloysite nanotubes can be performed by grafting –SO_3_H groups on their surface,^2,3,9^ or by treatment with acid solutions,^2–8,16^ including HCl,^4–7^ H_3_PO_4_,^16^ and a mixture of H_2_SO_4_–H_2_O_2_ (piranha solution),^8^ leading to an increase in both Lewis and Brønsted acidity.^4–8,16^

Although acidic halloysite nanotubes are active, selective, and stable catalysts for a number of organic reactions,^2–10^ to the best of our knowledge, the catalytic of the properties the materials based on their synthetic kaolinite analogues have not been reported. Moreover, natural halloysite nanotubes have different sizes and may contain impurities of other minerals; therefore, their physicochemical properties can vary significantly depending on the deposit.^1–3^ It can be expected that their synthetic analogues will have more constant composition and properties, which makes them promising materials for further functionalization.

**Fig. 2:**
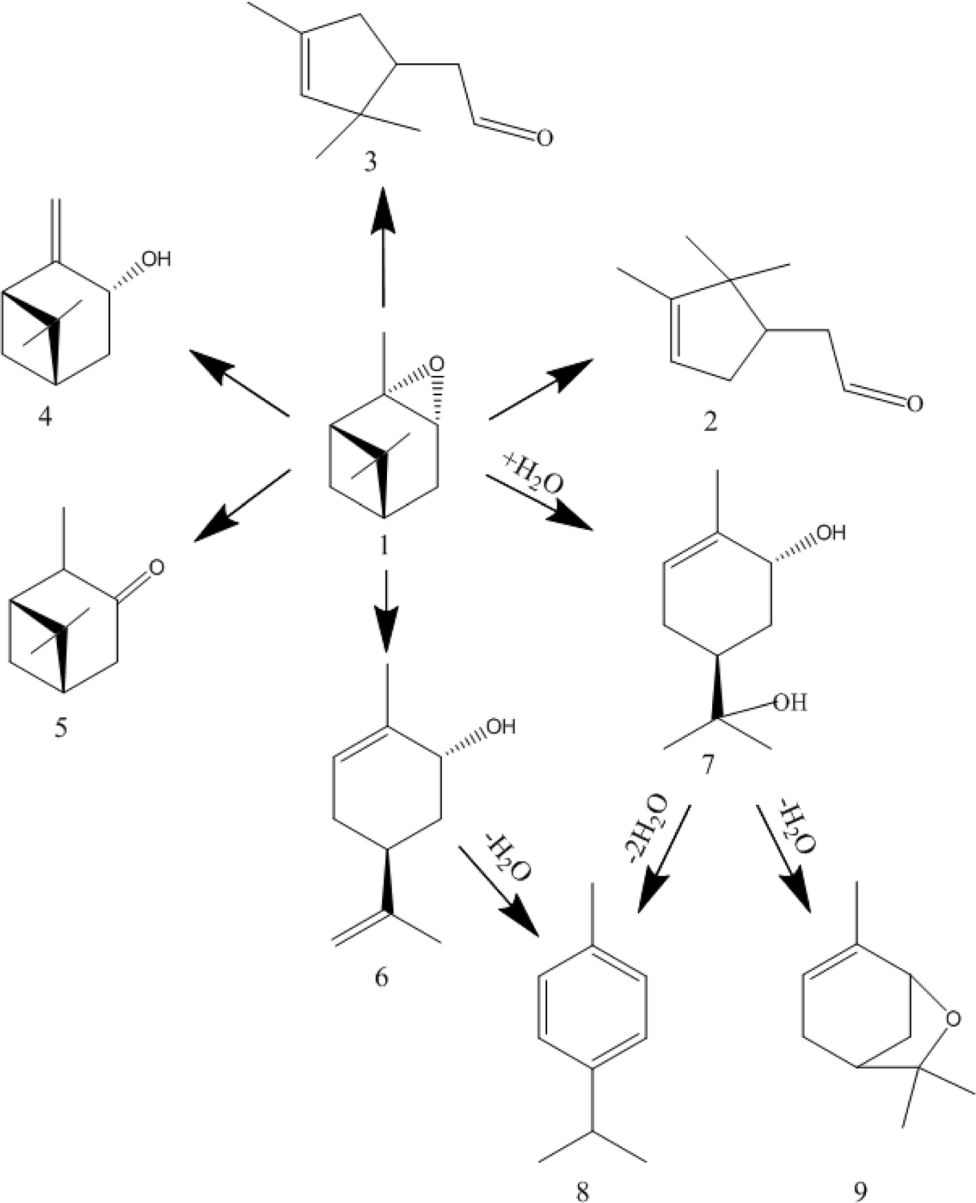
A general scheme of the catalytic isomerization of α-pinene epoxide (1) into campholenic aldehyde (2), iso-campholenic aldehyde (3), pinocarveol (4), isopinocamphon (5), *trans*-carveol (6), *trans*-sorberol (7), *p*-cymene (8) and pinol (9) (reproduced from ref. 21 with permission).

Epoxides of some terpene compounds are of interest for perfumery and fine synthesis,^17–20^ for example, terpene hydrocarbon α-pinene is the main component of turpentine (up to 85%) and is used in the production of a wide range of chemical products.^17–19^ Thus, during its mild oxidation, α-pinene oxide is formed, which is isomerized on an industrial scale into campholenic aldehyde, a precursor to a number of fragrant compounds.^17–19^ The general scheme of isomerization of α-pinene oxide is presented in Fig. 2.^21^ Traditional catalysts for this reaction are Lewis acids (ZnCl_2_, ZnBr_2_, *etc.*), however, in the recent years, novel heterogeneous systems have been proposed for the synthesis products based on α-pinene oxide, with the catalytic data summarized in the recent reviews.^17–19^

For example, selective formation of campholenic aldehyde (up to 96%) was observed on Ti-MCM-22 containing Lewis acid sites (tetrahedrally coordinated Ti species).^22^ The same high yield of this product (95%) was observed in the presence of Mo^IV^ complexes in ionic liquids.^23^ Quite high selectivity to campholenic aldehyde (82%) can also be achieved on Cu/MCM-41 at 70 °C in toluene.^24^ The mechanism of campholenic aldehyde formation in the presence of Fe^3+^ ions (up to 65%) was also studied by DFT computation in ref. 25. On layered aluminosilicates with weak acidity, the formation of almost equal amounts of campholenic and iso-campholenic aldehydes (68% in total) was observed.^21^

Another valuable product of α-pinene oxide isomerization is *trans*-carveol, which is part of essential oils used in the production of flavors and food components, and also exhibits anticancer activity.^17–19^ While the formation of campholenic aldehyde occurs mainly at the Lewis acid sites in non-polar solvents (toluene, benzene, *etc.*), the synthesis of *trans*-carveol occurs in polar basic media (*N*,*N*-dimethylformamide, *N*,*N*-dimethylacetamide *etc.*) especially on catalysts with the Brønsted acidity.^17–19^

Thus, high selectivity to *trans*-carveol (up to 93%) was observed in the presence of heteropoly acid H_3_PW_12_O_40_ in *N*,*N*-dimethylformamide.^26^ Carbon materials functionalized with acid groups, in particular biochar^27^ and microspheres,^28^ have shown high efficiency in the selective isomerization of α-pinene epoxide to carveol (up to 85%) in the same solvent. In ref. 29 phosphorus anion-based ionic liquids having alkyl ammonium, pyridinium or lutidinium cations were utilized for synthesis of *trans*-carveol with 74% selectivity at 99% substrate conversion. In the presence of hierarchical beta zeolites, a carveol yield of up to 42% was observed in *N*,*N*-dimethylacetamide at 140 °C on materials with well-developed mesoporosity and a high fraction of Brønsted acid sites of weak and medium strength.^30^

The purpose of this work is to (i) develop efficient method for the acid functionalization of kaolinite nanotubes (KNT), and (ii) compare their textural, acidic and catalytic properties (in isomerization of α-pinene oxide) with those of modified natural halloysite nanotubes.

## 2. Materials and methods

### 2.1. Synthesis and characterization of materials

Uzbekistan kaolins from Sulton Uvays, Alyans and Angren (grades AKF-78 and AKS-30) mines were used as raw materials. The main attention was paid to commercial kaolin AKF-78 due to its availability. To clean the surface from inorganic impurities, which facilitates further intercalation,^31^ kaolin samples were washed with 5% HCl. In a three-neck flask 30 g of clay and 150 mL of an acid solution were placed, heated to 80 °C and stirred at this temperature for 1 h. The precipitate was separated and washed with distilled water on a filter until the absence of Cl^−^, dried for 3 h at 105 °C and ground into powder. Halloysite nanotubes (HNT) were purchased from Sigma-Aldrich (origin Dragon Mine, USA).

The following typical procedure was used to synthesize aluminosilicate nanotubes.^15^ To a three-neck flask equipped with a mechanical stirrer, a thermometer, a reflux condenser, 10 g of kaolin, 60 mL of DMSO and 10 mL of distilled water were added. The contents were heated to 90 °C and stirred at this temperature for 6 h. Then the solid phase was separated on a filter and dried for 3 h at 60 °C, resulting in the DMSO form of kaolinite.

A sample of the resulting powder (3 g) in a filter paper sleeve was placed in a Soxhlet apparatus, into the cube of which 100 mL of methanol was poured and the treatment was carried out for 12 h with a duration of one extraction cycle of 15–20 min. The resulting MeOH–kaolinite was dried for 24 h at room temperature.

The MeOH–kaolinite was transferred to a three-neck flask, 100 mL of a methanol solution of CTACl (concentration 0.1 or 1.0 mol L^−1^) was added, heated to boiling (66 °C) and stirred at this temperature for 24 h using a magnetic stirrer. The solid phase was then separated, washed four times with hot ethanol, dried at 150 °C for 3.0 h. As a result, a material containing kaolinite nanotubes (KNT) was obtained.

Acid functionalization of kaolin nanotubes was carried out with a piranha solution (a mixture of 95% H_2_SO_4_ and 30% H_2_O_2_, volume ratio 3 : 1), choice of which was due to the need for (i) removal of organic impurities from them and (ii) acid functionalization. A sample of KNT (1.0 g) was placed in a three-neck flask, 5.0 mL of piranha solution was added and slowly heated to 40–50 °C, when gas evolution began. After destruction of the impurities, the mixture was heated to 90 °C and stirred at this temperature for 1 h. Then the solid phase was filtered, thoroughly washed with distilled water until acids and hydrogen peroxide were absent from the washing, dried (150 °C, 3.0 h) and fractionated (100 μm). This solid was designated as KNT-Pir.

The treatment of halloysite nanotubes was carried out according to a similar procedure, but only at 90 °C for 1 h due to the absence of organic impurities.

X-ray diffraction patterns of pretreated with HCl kaolinites were recorded on a Shimadzu XRD-6100XRD-6100 diffractometer (CuKα radiation, 5–70° 2*θ*). The chemical composition of AKF-78 samples was determined using EDX spectroscopy (JEOL JCM-6000Plus).

The porous structure of solids was measured with nitrogen physisorption on ASAP 2020 MP (Micromeritics) analyzer. The samples (*ca.* 50 mg) were previously evacuated (residual pressure 0.013 Pa) for 1 h at 200 °C. The specific surface area was calculated by the Brunauer–Emmett–Teller equation.^32^ The volume and the average diameter of the pores were determined from the desorption branch of the isotherm using the Barrett–Joyner–Halenda method.^33^ The chemical composition of the samples was determined by energy dispersive X-ray spectroscopy (EDX) using an electron microscope with a JEOL JCM-6000Plus chemical analysis system (Japan).

FTIR spectra of the samples were recorded in the region of 4000–400 cm^−1^ on a Bruker Tensor 27 spectrometer. Images solids obtained from kaolin were recorded using (i) a Zeiss Leo 1530 scanning electron microscope, as well as (ii) a LEO 906E transmission electron microscope. For the last one the samples were preliminarily dispersed in a water–ethanol mixture (3 : 1), applied to grids and secured by sputtering a carbon film.

The acidity of the catalysts was measured by infrared spectroscopy (Shimadzu, IRTracer-100) using pyridine as a probe molecule. A thin self-supported wafer of the catalyst (10–30 mg) was pressed and then placed into the FTIR cell. The catalyst wafer was outgassed at 350 °C for 1 h. Thereafter, the temperature was decreased to 100 °C and background spectra was recorded. Pyridine was adsorbed on the sample for 30 min at 100 °C followed by desorption at 150, 250 and 350 °C for 1 h and the spectra of the sample were recorded in between every temperature ramp. The scanning was performed under vacuum at 100 °C. Spectral bands at 1545 and 1450 cm^−1^ were used to identify Brønsted and Lewis acid sites, respectively. The quantitative amount of the acid sites was calculated using the extinction coefficient of Emeis.^34^

Note that the physicochemical properties of the parent and acid-treated HNT were carefully studied by XRD, EDX, ^27^Al and ^29^Si MAS NMR, SEM, TEM, FTIR with pyridine, and N_2_ adsorption and thermal analysis methods.^4^

### 2.2. Catalytic tests

To carry out isomerization of α-pinene oxide, 0.5 g of this compound was added to a two-neck flask (50 mL) equipped with a thermal controller and a magnetic stirrer, then 10 mL of the solvent was added. After heating the contents to the required temperature, 25 mg (5.0 wt%) of solid was added there and stirring was carried out (300 rpm), periodically taking samples for analysis. The composition of the reaction mixture was determined by gas–liquid chromatography using a Khromos GKh-1000 equipment with a flame ionization detector and a Zebron Zb-5 capillary column according to the method described in ref. 21.

## 3. Results and discussions

### 3.1. Physicochemical characteristics of KNT and comparison with HNT

**Fig. 3:**
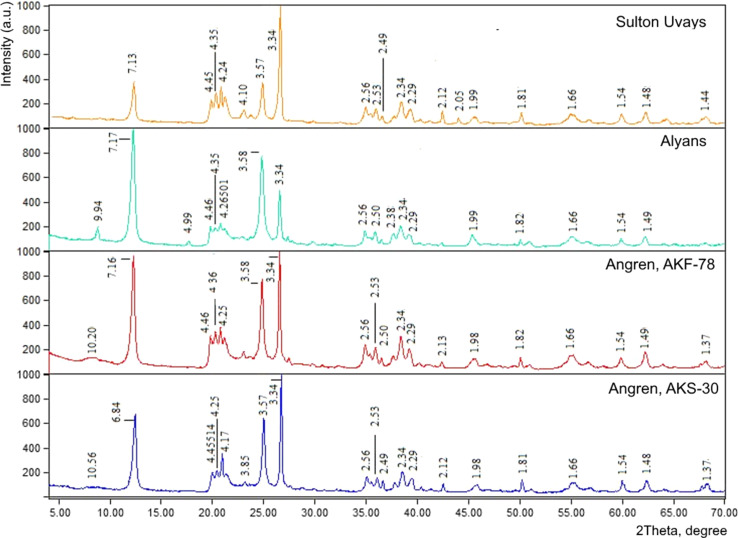
X-ray diffraction patterns of the studied kaolin from different deposits.

The diffraction patterns of kaolin clays washed with 5% HCl (Fig. 3) show reflections characteristic of kaolinite with interplanar distances of about 7.0 and 3.6 Å.^10,11^ Peaks around 10.0 and 3.3 Å indicate the presence of illite and quartz as impurities in all samples.^21^ The highest reflection intensity at 7 Å is characterized by Alyans and AKF-78 kaolins (Fig. 3).

**Fig. 4:**
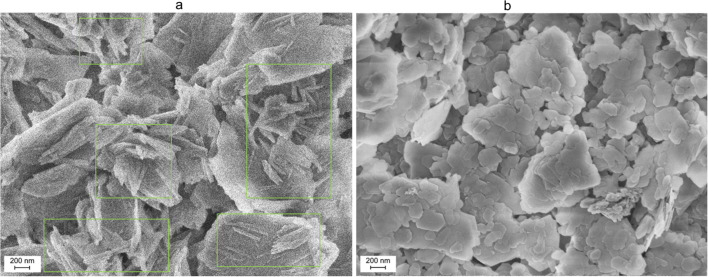
SEM images of the material obtained by sequential treatment of kaolin from Sulton Uvays (a) and Alyans (b) mines with DMSO, methanol and CTACl (0.1 mol L^−1^).

**Fig. 5:**
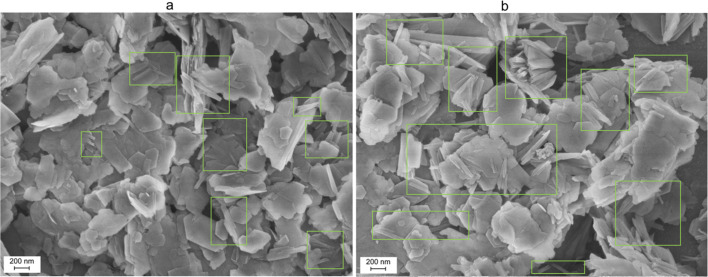
SEM images of the material obtained by sequential treatment of commercial kaolin AKC-30 (a) and AKF-78 (b) with DMSO, methanol and CTACl (0.1 mol L^−1^).

The synthetic procedure was initially implemented using a 0.1 mol per L CTACl solution in methanol. Scanning electron microscopy (SEM) images of Sulton Uvays and Alyans kaolins (Fig. 4) obtained after such treatment contain plate-like particles characteristic of kaolinite, and in the case of the first sample, the particles with tubular morphology are also observed (Fig. 4a). Treatment of commercial AKC-30 and AKF-78 under similar conditions also resulted in the formation of nanotubes, with the largest number of nanotubes found in the AKF-78 sample (Fig. 5b). However, according to SEM, unconverted kaolin predominates in all samples (Fig. 4 and 5).

**Fig. 6:**
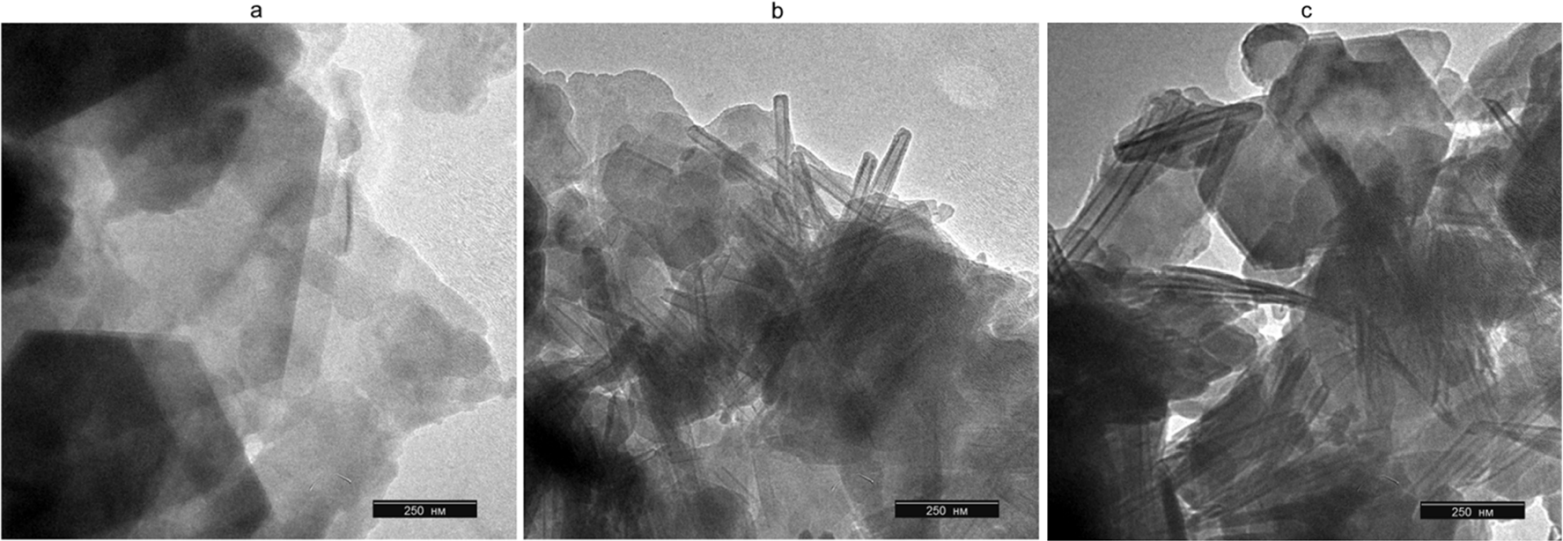
SEM images of kaolin AKF-78 (a), KNT obtained from it (CTACl 1.0 mol L^−1^, (b)) and the materials treated with piranha solution (KNT-Pir, (c)).

Further synthesis of KNT was carried out on the basis of commercial clay AKF-78, using a more concentrated CTACl solution (1.0 mol L^−1^), similar to the procedure described in ref. 15. According to the transmission electron microscopy image, under these conditions, nanosized tubes with a length of 600–1000 nm and a diameter of 15–25 nm were formed (Fig. 6b). At the same time, particles of the parent kaolin were also observed in small quantity.

**Fig. 7:**
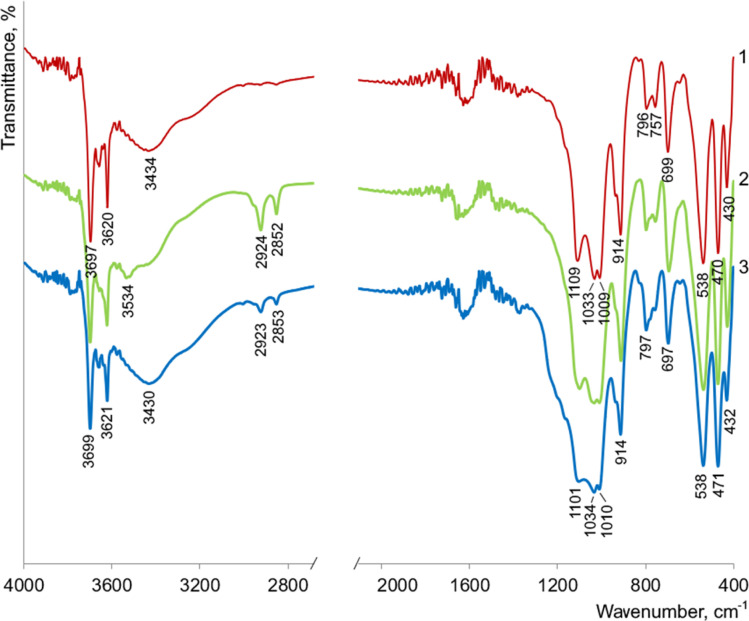
FTIR spectra of kaolinite AKF-78 (1), KNT obtained from it (2) and KNT-Pir (3).

The FTIR spectrum of the parent kaolinite AKF-78 (Fig. 7) shows intense absorption in the region of 1200–1000 cm^−1^ with peaks at 1109, 1033 and 1009 cm^−1^, which correspond to stretching vibrations of Si–O in its structure.^10,11,15^ A set of bands in the region of 600–400 cm^−1^ is associated with bending Si–O vibrations, among which the lines at 538 and 470 cm^−1^ belong to the Si–O–Al and Si–O–Si fragments, respectively.^10,11,15^ Absorption at 914 cm^−1^ is due to bending vibrations of Al–OH groups.^10,11,15^

In the region of 3700–3500 cm^−1^, the lines are observed at 3620 and 3697 cm^−1^, which are caused by stretching vibrations of structural –OH groups (Fig. 7). The last band is associated with vibrations of hydroxyls, near the surface of the octahedral (Al–O) layers of kaolinite and capable of forming hydrogen bonds with the tetrahedral (Si–O) layers.^10,11,15^ A broad absorption around 3434 cm^−1^ indicates the presence of adsorbed water molecules on the kaolinite surface.^10,11,15^

The FTIR spectrum of nanotubes obtained from AKF-78 is almost identical to that of the starting material (Fig. 7), indicating that the elements of the kaolinite structure are preserved. The doublet that appears at 2924 and 2852 cm^−1^ (–CH_2_– and CH_3_–) clearly indicates the presence of organic compounds in the resulting nanotubes.^10,11,15^ The broad line in the region of 3434 cm^−1^, observed for AKF-78, sharply decreases in intensity and shifts to 3534 cm^−1^, which indicates a significant decrease in the content of adsorbed water in KNT, apparently also due to organic impurities. Such impurities are typical for kaolinite nanotubes and remain as a result of incomplete removal of reagents after the synthesis procedure.^10,11,15^

As a result of the kaolinite nanotubes treatment with a piranha solution, a significant decrease in the intensity of the peaks around 2923 and 2853 cm^−1^ occurs in their FTIR spectrum, while absorption in the 3600–3400 cm^−1^ region (3430 cm^−1^, H_2_O) sharply increases in intensity, becoming close to that of the original kaolin AKF-78 (Fig. 7). Based on this, it can be assumed that such treatment leads to the removal of a significant number of organic impurities and an increase in the hydrophilicity of the nanotube surface. Noted that the shape and intensity of the lines at 538, 1034 (Si–O), 3621 and 3699 cm^−1^ (–OH) do not change, which clearly indicates the preservation of the crystalline structure of the nanotubes after exposure to the H_2_SO_4_–H_2_O_2_ mixture. This is also clearly confirmed by the TEM image of KNT-Pir (Fig. 6c), according to which the shape and size of the nanotubes did not change.

The composition of the parent kaolinite AKF-78 and halloysite includes mainly Al_2_O_3_ and SiO_2_, as well as small amounts of oxides of iron, sodium, magnesium, potassium, calcium, and titanium (Table 1). A higher SiO_2_ content in AKF-78 originated from the presence of quartz as an impurity, as evidenced by XDR data (Fig. 3). The commercial halloysite used in this work practically does not contain any impurities.^4^ Note that in the process of obtaining nanotubes from kaolinite, a change in the composition of inorganic substances should not occur, since the synthetic procedure is aimed at changing the morphology of its particles,^10,11,15^ while organic impurities are present in the resulting KNT (Fig. 7).

**Table 1:** Chemical composition and porous structure of the investigated solids

Sample	Chemical composition, wt%								Porous structure		
	Al_2_O_3_	SiO_2_	FeO	Na_2_O	MgO	K_2_O	CaO	TiO_2_	*S*_BET_, m^2^ g^−1^	*V*_pore_, cm^3^ g^−1^	*D*_pore_, nm
Kaolin AKF-78	42.5	53.9	0.9	0.1	0.3	2.0	0	0.4	33	0.10	10.3
KNT	n.a.								41	0.15	12.1
KNT-Pir	30.9	66.0	0.8	0.1	0.2	1.4	0	0.7	159	0.23	10.6
HNT	49.0	49.5	0.7	0.1	0.1	0	0.3	0.4	60	0.22	15.7
HNT-Pir	39.7	59.2	0.5	0.1	0	0	0.3	0.3	165	0.46	11.7

Treatment of both KNT and HNT with the piranha solution leads to a decrease in Al_2_O_3_ content and an increase in the amount of SiO_2_ in the resulting samples (Table 1). This is because of the mechanism of layered aluminosilicates acid etching, which involves the breaking of Si–O–Al bonds between their tetrahedral silicon-oxygen and octahedral aluminum-oxygen layers, ultimately leading to leaching of Al^3+^ into the solution and formation of amorphous SiO_2_.^4,20,35^

**Fig. 8:**
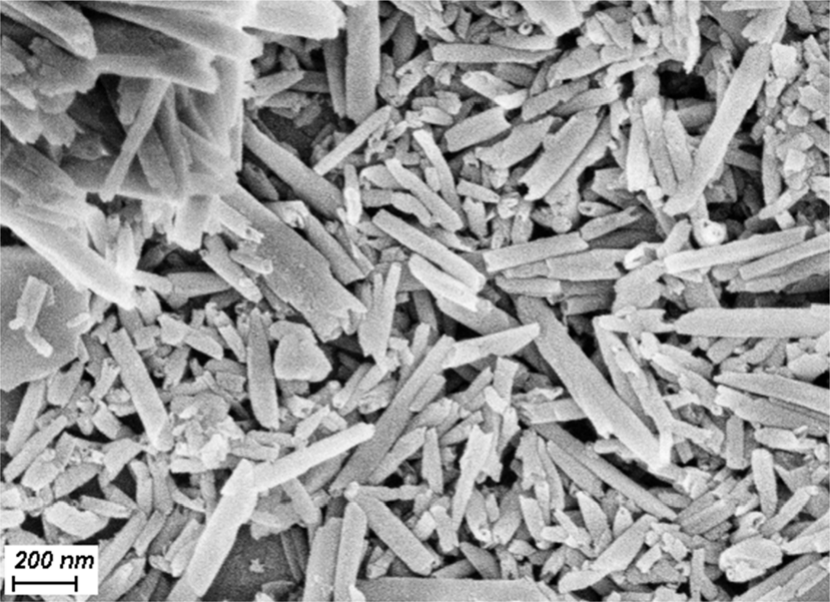
SEM image halloysite pretreated with the piranha solution (HNT-Pir).

The same as for KNT (Fig. 6b and c), treatment of HNT with a piranha solution does not lead to the destruction of nanotubes, since their shape and size (Fig. 8) correspond to those for Dragon Mine type halloysite.^4,36^ According to ref. 4, significant degradation of halloysite nanotubes occurs when more than 50% of the Si–O–Al bonds are destroyed.

The specific surface area (*S*_BET_) of the original AKF-78 kaolin equal 33 m^2^ g^−1^ was slightly increases for the nanotubes obtained from it (41 m^2^ g^−1^, Table 1). Note that an almost similar *S*_BET_ value (39 m^2^ g^−1^) was observed for KNT synthesized on the basis of commercial (Sigma-Aldrich) kaolin.^14^ For the parent halloysite, the specific surface area is also comparable (60 m^2^ g^−1^, Table 1).

Pretreatment of KNT and HNT with the piranha solution resulted in an increase in both *S*_BET_ and pore volume. At the same time, the specific surface area values for the acid-modified KNT/HNT-Pir materials are almost the same (159–165 m^2^ g^−1^). Activation of halloysite from China (Hubei) using a similar method led to an increase in *S*_BET_ to 73 m^2^ g^−1^.^37^ On the other hand, a higher surface area of HNT (up to 207 m^2^ g^−1^) was observed after their treatment with 30% HCl at 90 °C, which, however, caused almost complete destruction of the tubular structure.^4^

Recently,^38^ using the method of molecular dynamic modeling, it has been shown that during Al leaching, some nanoporous (1–20 nm) interlayer spaces are formed inside the wall of HNT due to the strong bonds between the adjacent layers. The interlayer nanopores greatly increase the specific surface area and the pore volume of halloysite, which is more prominent after 50% Al leaching. These conclusions correspond to the results obtained earlier in ref. 4 which during halloysite HCl treatment the highest values of *S*_BET_ and *V*_pore_ were after 50% removal of aluminum.

Worth noting that the activation of KNT with an HCl solution is ineffective, since in this case the formation of a gel was observed, apparently due to the interactions of the acid with the CTACl impurity, and the resulting material did not have any catalytic activity.

**Fig. 9:**
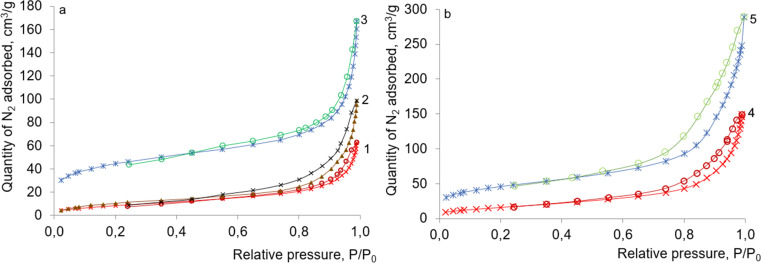
Nitrogen adsorption–desorption isotherms of (a) kaolinite AKF-78 (1), KNT obtained from it (2), KNT-Pir (3), and (b) HNT initial (4) and HNT-Pir (5).

Isotherms of low-temperature nitrogen adsorption–desorption for solids based on kaolin AKF-78 and halloysite are presented in Fig. 9. All of them belong to type IV(a), according to the IUPAC classification.^39^ Narrow capillary-condensation hysteresis loops on the isotherms belong to type H3 and have two distinctive features: (i) their adsorption branches resemble type II isotherms, and (ii) the lower boundary of the desorption branches is at the level of the relative cavitation pressure, *p*/*p*_0_ ∼ 0.42. Loops of the type H3 are formed, as a rule, by non-rigid aggregates of lamellar particles (usually in layered minerals). Thus, the obtained isotherms are typical for mesoporous materials; the average pore diameter calculated from their desorption branch is 10.3–15.7 nm (Table 1).

The concentration of acid sites (a.s.) on the AKF-78 kaolin surface is 15.0 μmol g^−1^, decreasing to 12.0 μmol g^−1^ for the nanotubes obtained from it (Table 2). The lower acidity value may be due to blocking of the active sites by organic impurities that are present in KNT (Fig. 7). Treatment of kaolinite nanotubes with a piranha solution leads to a sharp increase in the number of acid sites (49.0 μmol g^−1^), primarily Lewis ones. It is known that during the acid etching of layered aluminosilicates (including kaolinite, halloysite, montmorillonite, *etc.*), a cation exchange occurs leading to the formation of the H^+^ form, which is unstable and is converted spontaneously to the Al^3+^ form by the release of structural Al^3+^ cations onto the particles surface.^35,40^ An increase in the L/B ratio was also observed when illite clay was treated with HCl, while the total concentration of a.s. in the resulting catalyst (up to 47.0 μmol g^−1^ (ref. 21)) was the same as that for KNT-Pir (49.0 μmol g^−1^).

**Table 2:** Acidic properties of the investigated solids

Catalyst	Acid site concentration, μmol g^−1^							L/B
	Brønsted (B)			Lewis (L)			Total	
	Weak	Medium	Strong	Weak	Medium	Strong		
Kaolinite (AKF-78)	1.0	1.0	3.0	3.0	3.0	4.0	15.0	2.0
KNT	0	3.0	1.0	5.0	2.0	1.0	12.0	2.0
KNT-Pir	1.0	3.0	5.0	27.0	6.0	7.0	49.0	4.4
HNT^a^	12.0	1.0	0	17.0	4.0	0	34.0	1.6
HNT-Pir	30.0	10.0	0	12.0	7.0	0	59.0	0.5

a The data from ref. 4.

The amount of a.s. for the starting halloysite nanotubes is 34.0 μmol g^−1^, being significantly lower than for kaolin (15.0 μmol g^−1^, Table 2). Note that due to the almost complete absence of isomorphism in kaolinite and halloysite, the active sites should be located at the ends and faces of their particles.^4,35^ The higher dispersion of halloysite particles is obviously the reason for its larger acidity. Treatment of HNT with the piranha solution also leads to an increase in the number of a.s. up to 59.0 μmol g^−1^, which is slightly higher than in the case of KNT-Pir (49.0 μmol g^−1^). At the same time, the Brønsted type of acidity predominates in HNT (Table 2).

According to ref. 8, etching halloysite (Sigma-Aldrich) with the piranha solution gives the same acidity value (59.8 μmol g^−1^ with L/B 1.6, measured at 200 °C). The reasons for the low concentration of Brønsted a.s. in KNT-Pir compared to HNT processed under similar conditions require additional detailed studies.

Note that the treatment of HNT (Sigma-Aldrich) with HCl allows one to increase its acidity up to 52.0 μmol g^−1^,^4^ whereas in the case of H_3_PO_4_ its values are equal to 45.0 μmol g^−1^.^16^ In addition, effective methods for acid-functionalizing silicates, including halloysite nanotubes, is the fixation of –SO_3_H groups on their surface using various reagents (chlorosulfonic acid, 2-(4-chlorosulfonylphenyl)ethyltrimethoxysilane, ionic liquids, *etc.*) under elevated temperatures^3,9,41,42^ or an electron beam irradiation.^43^ Based on this, grafting of various functional groups on the surface of KNT after cleaning from organic impurities by the piranha solution is a promising direction for further work.

### 3.2. Catalytic activity of acid-functionalized HNT and KNT

For an initial screening of α-pinene oxide isomerization, halloysite treated by the piranha solution was used as a more accessible catalyst. The reaction was carried out in various solvents, including so-called green ones, since the reaction rate and selectivity are sensitive to the medium used.^17–19^

In the presence of non-polar solvents (cyclohexane, *p*-cymene), the isomerization of α-pinene oxide on HNT-Pir proceeded at a high rate and with an increase in the temperature of the reaction mixture (on 10–15 °C), which caused almost complete conversion of the substrate within 15 min of the reaction (Table 3). The main products of the reaction are campholenic and iso-campholenic aldehydes, which in the case of cyclohexane are formed in a total amount of 57.8%. Similar selectivity values were observed in the same solvent on pretreated by HCl illite clay with comparable acidity (47.0 μmol g^−1^).^21^

**Table 3:** Initial screening of catalytic activity of HNT-Pir^a^ in α-pinene oxide isomerization for 180 min in different solvents

Solvent	Temperature, °C	*r*_0_, mmol (g^−1^ min^−1^)	Conversion 1, mol%	Selectivity, mol%							
				2	3	4	5	6	7	8	9
Cyclohexane	30	Large	99.9^b^	32.4	25.4	0.6	1.3	12.5	8.6	5.5	2.1
*p*-Cymene		Large	99.9^b^	34.2	14.5	0.3	1.5	17.0	14.7	n.d.	2.6
Isopropyl acetate		1.5	95.0^c^	28.0	5.7	0.6	1.0	18.9	28.0	6.8	4.4
Dimethyl carbonate		1.0	42.8	51.2	5.7	1.0	3.6	18.3	15.0	4.3	1.5
Diethyl carbonate		1.1	50.4	34.4	7.0	0.8	1.4	26.5	22.0	5.8	2.6
Ethyl lactate		Large	99.9^d^	50.4	7.0	0.1	0.2	12.7	24.1	4.2	0.1
γ-Valerolactone		14.3	99.9^c^	25.7	2.0	0	0	18.5	45.8	5.9	0.5
2-Methyl-tetrahydrofuran		1.8	95.0	17.4	8.3	0.3	0	43.4	26.8	2.4	0.1
DMSO		No reaction									
	90	0.35	95.3	22.8	1.8	0.8	0	55.4	15.5	0.5	0
*N*,*N*-Dimethylform-amide (DMF)		No reaction									
	140	0.8	31.9	13.7	0.7	1.8	2.3	57.1	20.0	2.1	0.4
1 – α-pinene oxide, 2 – campholenic aldehyde, 3 – iso-campholenic aldehyde, 4 – pinocarveol, 5 – iso-pinocamphone, 6 – *trans*-carveol, 7 – *trans*-sorberol, 8 – *p*-cymene; 9 – pinol											

a Dried for 2 h at 105 °C.

b For 15 min of the reaction.

c For 2 h of the reaction.

d For 1 h of the reaction.

In the presence of polar solvents containing an ester group (isopropyl acetate, dimethyl and diethyl carbonates, ethyl lactate), the main reaction products were campholenic aldehyde 2 (up to 51.2%) and *trans*-sorberol 7 (up to 28.0%), while the content of isomeric aldehyde 3 was insignificant (Table 3). In these media, the initial reaction rate (*r*_0_) is lower than in hydrocarbons, however, in ethyl lactate, isomerization is completed within 1 h of the reaction.

In turn, a highly polar green solvent γ-valerolactone (dielectric constant *ε* 36.4 (ref. 44)) causes the reaction to proceed at a relatively high initial rate with the preferential formation of *trans*-sorberol (45.8%, Table 3). According to ref. 26, the formation of this compound (up to 71%) was observed in the presence of polar weakly basic solvents (acetone, acetonitrile).

*trans*-Carveol 6 predominates in the reaction mixture (43.4%, Table 3) when using 2-methyl-tetrahydrofuran, which can be an environmentally friendly replacement for tetrahydrofuran, in the presence of which α-pinene oxide isomerizes on MoO_3_/beta to form almost equal amounts of campholenic aldehyde and the product 6 (*ca.* 30%).^45^

When using highly polar basic solvents (DMSO and DMF), higher temperatures were required for epoxide isomerization (90 and 140 °C, respectively), while almost complete substrate conversion was observed when DMSO was used. Selectivity to *trans*-carveol in these media was approximately the same, being 55–57% (Table 3).

**Fig. 10:**
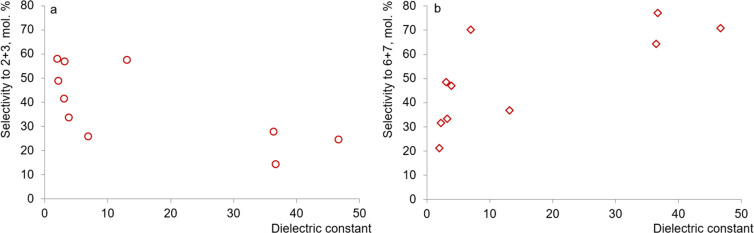
Correlation between the dielectric constant of the solvent and selectivity for aldehydes 2 and 3 (a) as well as alcohols 6 and 7 (b).

To quantify the effect of solvents on selectivity, a comparison was made between the dielectric constant of the medium (*ε*) and the lumped selectivity to the resulting aldehydes 2 and 3 (Fig. 10a), as well as alcohols 6 and 7 (Fig. 10b). Thus, with increasing solvent polarity, a decrease in selectivity for products 2 and 3 is observed, the largest amount of which is formed in the presence of non-polar cyclohexane (*ε* 2.02). On the other hand, the selective formation of alcohols 6 and 7 is observed in the presence of polar media (DMSO, DMF, γ-valerolactone) with high *ε* values of 36.4–46.7. The relatively high lumped yield of products 6 and 7 when using weakly polar (*ε* 6.97) 2-methyltetrahydrofuran may be associated with its relatively high basicity.^45^

Comparison of the catalytic properties of KHN-Pir and HNT-Pir was carried out in cyclohexane, ethyl lactate and DMSO, since a high substrate conversion was observed on halloysite in these solvents, and the reactions proceeded with different selectivity values.

Kaolin AKF-78, as well as KNT synthesized from it, did not have any catalytic activity in the isomerization of α-pinene oxide in cyclohexane. The KNT/HNT-Pir materials in this solvent showed similar selectivity, with isomeric campholenic aldehydes as the main products (Table 4). At the same time, the reaction rate on KNT-Pir was lower, and for 99% conversion of the substrate required 3 h (Table 4), which may be due to the lower nature and concentration of a.s. in KNT-Pir (Table 2). Similar results were previously observed on modified by HCl illite clay, where Lewis a.s. predominated at a high degree of dealumination (L/B up to 6.7), which caused a decrease in the reaction rate, but the selectivity did not change significantly.^21^

**Table 4:** Catalytic activity of KNT-Pir and comparison with HNT-Pir in α-pinene oxide isomerization for 180 min

Catalyst^a^	Solvent	*r*_0_, mmol (g^−1^ min^−1^)	Conversion 1, mol%	Selectivity, mol%							
				2	3	4	5	6	7	8	9
Kaolin	Cyclohexane	No reaction									
KNT											
KNT-Pir		2.9	99.0	41.4	22.7	0.7	1.8	16.4	9.8	5.1	1.3
HNT-Pir		Large	99.9^b^	32.4	25.4	0.6	1.3	12.5	8.6	5.5	2.1
KNT-Pir	Ethyl lactate	0.87	24.0	54.7	6.3	1.3	0	12.7	20.3	3.4	0.1
HNT-Pir		16.8	99.9^c^	50.4	7.0	0.1	0.2	12.7	24.1	4.2	0.1
KNT-Pir	DMSO	0.30	97.2	22.5	2.4	0.8	0	56.8	13.3	0.6	0.2
HNT-Pir		0.35	95.3	22.8	1.8	0.8	0	55.4	15.5	0.5	0
1 – α-pinene oxide, 2 – campholenic aldehyde, 3 – iso-campholenic aldehyde, 4 – pinocarveol, 5 – iso-pinocamphone, 6 – *trans*-carveol, 7 – *trans*-sorberol, 8 – *p*-cymene; 9 – pinol											

a Dried for 2 h at 105 °C.

b For 15 min of the reaction.

c For 1 h of the reaction.

In the case of ethyl lactate, the reaction on KNT-Pir proceeded at a significantly lower rate, and the substrate conversion after 3 h was only 24%, which contrasts with the halloysite activity, where the reaction was completed after 1 h of the reaction. This may be due to catalyst deactivation of the catalyst, probably due to the interactions of the active Lewis sites in KNT-Pir with the hydroxyl group of the solvent.

When using DMSO as a solvent, isomerization of α-pinene oxide occurs efficiently at 90 °C on aluminosilicate nanotubes of both types (KNT/HNT-Pir), leading to formation of *trans*-carveol 6 with almost the same selectivity (55–57%). A similar yield of product 6 (53.6%) was observed in the presence of MoO_3_/beta catalyst in the same solvent.^45^

Note that when using toxic solvents such as *N*,*N*-dimethylformamide and *N*,*N*-dimethylacetamide, selectivity towards *trans*-carveol was 43–44% on MoO_3_/beta at 70 °C,^45^ 50.6% in the presence of ammonium phosphotungstate on activated carbon^46^ and 42% on hierarchical beta zeolites at 140 °C.^30^ The KNT-Pir and HNT-Pir catalytic systems in low-toxicity solvent, DMSO, can be used for the efficient isomerization of α-pinene oxide to valuable *trans*-carveol in the context of green chemistry.

**Fig. 11:**
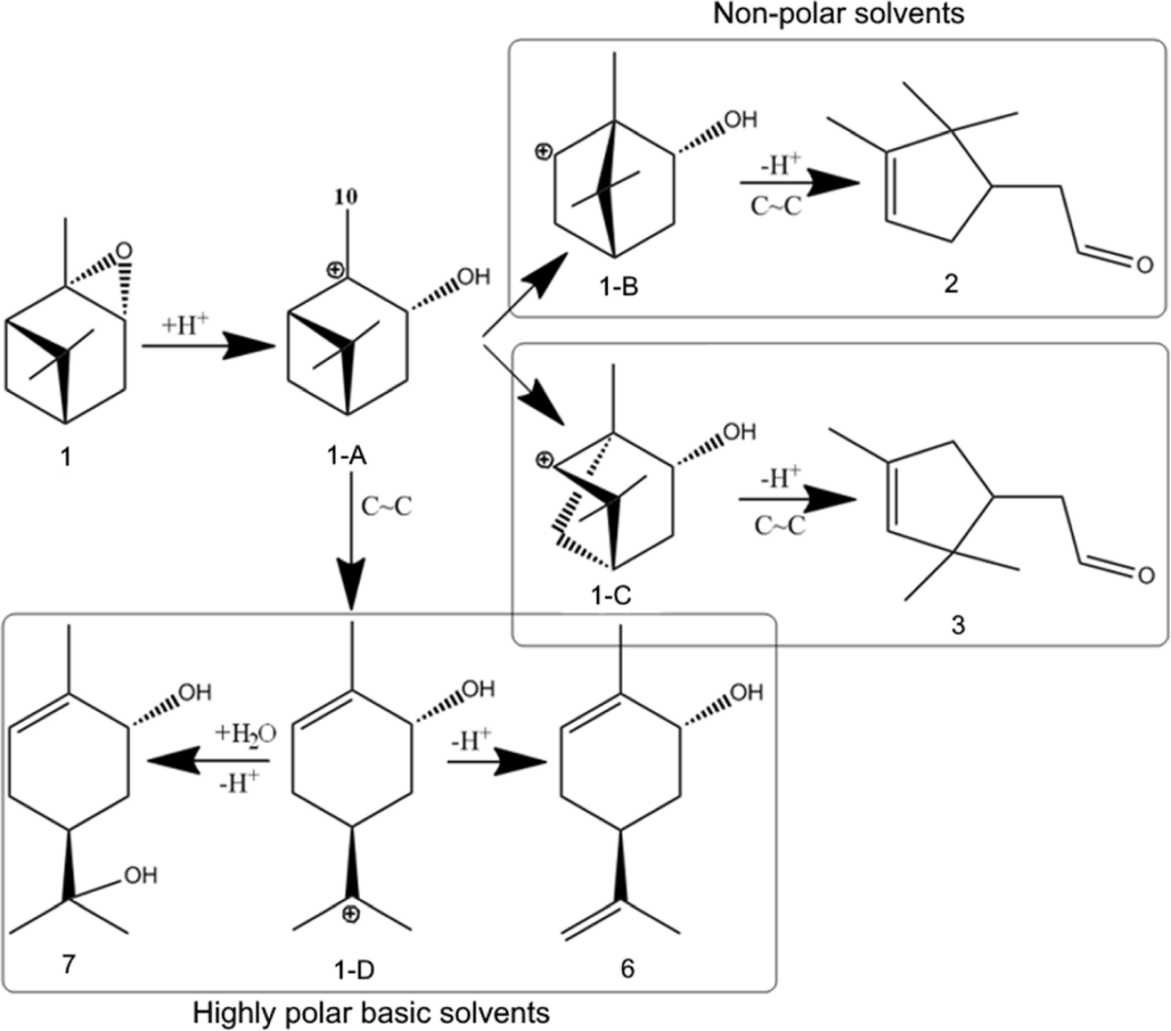
Mechanism of α-pinene oxide isomerization.

The acidic properties of aluminosilicate nanotubes, in particular, halloysite, are due to the presence of Brønsted (H^+^, acidic –OH groups, polarized water molecules) and Lewis (exchange and structural Al^3+^ ions) species on their surface.^4,35,37^ The Brønsted character of the clay acidity prevails at drying temperatures up to 200 °C due to the presence of adsorbed water on the surface, and a temperature increase leads to predominance of the Lewis sites.^47^ Based on drying HNT/KNT-Pir at 150 °C before the reaction, the Brønsted a.s. should determine their catalytic activity, therefore the mechanism of the studied reaction (Fig. 11) is considered from the point of view of protons as the active site.

At the first stage, protonation of the substrate occurs with the formation of the 1-A carbocation, further transformations of which occur in several directions. Thus, when the cyclobutane ring of the 1-A ion expands, intermediates 1-B and 1-C can be formed, which leads to the formation of campholenic 2 and iso-campholenic 3 aldehydes, respectively. Such reactions preferably occur in nonpolar solvents on weakly acidic catalysts,^21^ as in the present case. Selective formation of campholenic aldehyde, *i.e.* when the transformation of 1-A predominantly into 1-B occurs on catalysts with Lewis acidity and a relatively high concentration of a.s.^22–25^

Another direction of 1-A transformation is the opening of the cyclobutane ring to form the carbocation 1-D, which can give *trans*-carveol 6 and *trans*-sorberol 7 as products (Fig. 11). Relatively selective formation of compound 6 occurs in the presence of polar basic solvents on catalysts with Brønsted^26–29^ or both types of acidity.^30^ In the present work, this product was also formed in the presence of solids containing both Lewis and Brønsted centers. Isomerization of α-pinene oxide is considered as a reaction in which formation of the main products (campholenic aldehyde, *trans*-carveol) proceeds in a parallel course.^21,30,48^ The kinetic and thermodynamic aspects of these transformations have been thoroughly studied in the literature.^29,30,48,49^

Overall, this work is the first example of the utilization of functionalized synthetic kaolinite nanotubes as a catalyst. Future work may include investigation of various methods for their modification, including grafting of functional groups and testing of catalytic activity.

## 4. Conclusion

Kaolinite nanotubes (KNT) were obtained by sequentially treating commercial kaolin AKF-78 (Uzbekistan) with dimethyl sulfoxide, methanol, and a solution of cetyltrimethylammonium chloride. The materials were characterized by EDX, XRD, FTIR, N_2_ adsorption–desorption methods. After synthesis, nanotubes contain organic impurities, which complicates their further functionalization and use as catalysts. This drawback was successfully eliminated by two-stage treatment of KNT with a piranha solution (H_2_SO_4_–H_2_O_2_), where at the first stage organic impurities are effectively removed, and at the second the parameters of the porous structure (159 m^2^ g^−1^, 0.23 cm^3^ g^−1^) and acidity (49 μmol g^−1^) of the nanotubes increased. The achieved parameters were close to those for halloysite nanotubes (HNT) treated with the same reagent. Both kaolin and halloysite nanotubes treated with H_2_SO_4_–H_2_O_2_ show catalytic activity in the isomerization of α-pinene epoxide model reaction in various solvents, including so-called green ones. In non-polar media (cyclohexane) the formation of mainly campholenic aldehydes occurred, while polar basic solvents (DMSO, DMA) gave *trans*-carveol as the main product (55–57%). In general, treating KNT with a piranha solution is a simple and effective way to purify and modify them, allowing to increase porosity and acidity, which opens the way for further functionalization and practical use of these nanomaterials.

## Data availability

The obtained data are provided in the manuscript.

## Conflicts of interest

There are no conflicts to declare.
